# Different Types of Physical Activity and Fitness and Health in Adults: An 18-Year Longitudinal Study

**DOI:** 10.1155/2017/1785217

**Published:** 2017-03-29

**Authors:** Steffen C. E. Schmidt, Susanne Tittlbach, Klaus Bös, Alexander Woll

**Affiliations:** ^1^Institute of Sport and Sport Science, Karlsruhe Institute of Technology, Karlsruhe, Germany; ^2^Institute of Sport Science, University of Bayreuth, Bayreuth, Germany

## Abstract

*Objective*. The aim of this study is to examine the relationship between different types of daily life physical activity (PA) and physical fitness (PF) and health throughout adulthood.* Methods*. A total of 723 men and women, aged 28–76 years, participated 1681 times during four measurement points from 1992 to 2010 in this study. We assessed self-reported PA, anthropometrics, physical health status (HS), and PF in each study year. Hierarchical linear modeling (HLM) was used to analyze the measures.* Results*. PF and HS worsened with increasing age while sports activity (SA) declined. The modeling showed that sex, age, and SES play important roles concerning PA, PF, and HS. Athletes show higher HS and HF than nonathletes. Habitual activity (HA) also showed a positive relationship with PF and HS, but effects were lower than for SA. Work related activity (WRA) showed no meaningful relationship with PF or HS.* Conclusions*. Comparable amounts of PA can lead to different effects on PF or HS. Our findings underline the importance of contexts, content, and purposes of PA when health or fitness benefits are addressed. Simply moving your body is not enough.

## 1. Introduction

**Figure 1:**
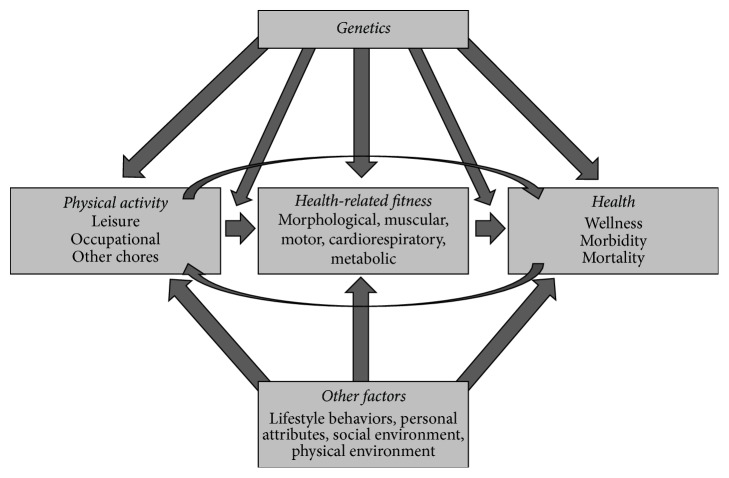
Relationship between PA, fitness, and health [6].

There is consensus that regular physical activity (PA) can improve physical fitness (PF) and health and assist in the prevention of disease [1, 2]. Several studies have shown that physically active adults are healthier and have a higher PF than inactive adults throughout different nations and populations groups [3, 4]. Physical activity is therefore promoted as part of a healthy lifestyle [5]. The current understanding of the relationship between PA, PF, and health can be visualized using the model from Bouchard et al. [6] (Figure 1). The model illustrates that PA can influence fitness and health and that the relationships are also reciprocal. Additionally, other factors such as personal and social attributes age, sex, and socioeconomic status (SES) are known to influence PF, HS, PA, and their relationships.

Besides commonly known positive effects of PA, it is also known that the relationship between PA and PF and health varies between different amounts, intensities, and contexts of physical activity and a clear dose-response principle between amount, intensity, and effect is yet not known [7, 8]. For example, recent studies that assess work related activity fail in finding a positive influence on body composition and health factors [9, 10] and especially in the elderly, injuries and physical wear and tear caused by PA are not uncommon [11]. Nevertheless, from randomized controlled studies we know that applied “high quality” PA that is planed PA in controlled circumstances can improve fitness and health in every stage of life [12]. However, the state of research concerning longitudinal effects of nonapplied, long-term, daily life PA such as habitual activity for transportation, long time sports club activity, or work related activity is lacking. A meta-analysis by Dionne et al. [4] described six studies with high methodological quality about the relationship between daily life PA and cardiovascular fitness and the reported correlations ranged from *r* = .25 to *r* = .76. Other authors suggest that the relationship between PA and health and PF measures strongly depends on sociodemographic characteristics (e.g., age, sex, and SES), settings (e.g., leisure time PA, commuting, and sports), extent of physical activity (intensity, frequency, and duration), and fitness level as well as on the health and fitness measures [13].

In order to analyze the relationship between different types of long-term PA, PF, and health throughout the lifespan, laborious longitudinal studies are needed. However, most of the conducted longitudinal studies refer to effects of physical activity on very specific health diseases, such as type 2 diabetes mellitus [14], depression [15], osteoporosis [16], or chronic pulmonary disease [17] or focus only on trends of PA [18] fitness and health [19]. In addition few have considered the dependency on demographic factors (e.g., age, sex, and socioeconomic status).

Therefore the aim of this study is to examine the longitudinal relationship between different types of nonapplied, daily life PA and PF and HS in adults and to assess the influence of sociodemographic determinants age, sex, and SES.

## 2. Research Methods

### 2.1. Study Sample and Design

The data was drawn during a community-based, longitudinal study in Germany [20] with four measurements in 1992, 1997, 2002, and 2010. Participants were randomly selected from the local residents' registration offices. Participation was voluntary. Subjects provided their written consent to participate in the study. The applied protocols were approved by a scientific advisory council, the Schettler Clinic, Bad Schönborn, Germany, as well as the ethic committee of the Karlsruhe Institute of Technology (KIT).

A total of 723 different subjects (366 f and 357 m) aged 28–76 participated 1681 times over the course of the study. The response rate of the initial sample in 1992 was 56%. For the initial sample, five groups of 35, 40, 45, 50, and 55 ± 2 years old were invited. In each subsequent wave, new participants from 28 to 38 were recruited to compensate for drop outs. The total number of participants for each of the four measurement points was 1992: 480, 1997: 456, 2002: 429, and 2010: 310. A nonresponder telephone interview showed no significant differences in selected parameters (e.g., SES, physical health status, and physical activity) between participants and invited nonparticipants except migration background [21]. Descriptive statistics of the sample are shown in Table 1.

The sample shows representative characteristics regarding BMI and SES for a rural community in Germany. PA however tends to be slightly over average for Germany [22].

### 2.2. Measures

#### 2.2.1. Physical Activity

Weekly sports activity, habitual activity, and work related activity were assessed via questionnaire. An estimation of the weekly energy expenditure in MET-hours per week for SA, HA, and WRA was calculated according to Ainsworth et al. [23] as a product of weekly frequency, duration, and intensity of the type of activity.

Sports activity (SA) was calculated from questions about frequency (number of weekly exercise units), duration (minutes per unit), intensity (not very intense, moderate intense with some sweating, and highly intense with much sweating), and type of weekly sports activity [24]. For each of the three intensities, every type of sport was assigned a specific MET value [23] and by multiplication with the spent time, SA in MET-hours per week was calculated. Habitual activity (HA) was derived from daily times of walking and biking for transportation as well as working in the household and gardening. Again, each type of HA was allocated a specific MET value according to Ainsworth et al. [23] and MET-hours per week were calculated. Work related activity (WRA) was derived from time spend at work, a question about type of activity at work (mainly sitting, mainly standing, mainly walking, and/or being in movement), and a question about intensity of activity at work (not very intense, moderate intense, and high intense). MET-hours per week for WRA were then calculated using the respective METs for work place activity [23].

A priori analyses showed that, in addition to the amount of physical activity, a dichotomous variable made of the question “Do you exercise? Yes/no” significantly improved the model fits. In addition to the amount of SA, a variable “athlete” was included in the models that separates between participants that exercise and participants who completely call themselves unsporting. It stands for effects of an active lifestyle which are not dependent on the amount of exercise. In addition, the following stratification was used in the figures: “no sport”: participant who continuously reported no SA; “sport quitters”: participants who reported SA at their first but not on their last examination; “sport beginners”: participants who reported no SA at their first but on their last examination; “continuous athletes”: participants who reported SA on each examination. The questionnaire was proofed for reliability (test-retest after two weeks: *r* > .90 and Cronbach*ʼ*s alpha = .94), factorial validity, and measurement invariance [25].

#### 2.2.2. Physical Fitness (PF)

In total 13 motor performance tests were used to assess physical fitness [26]. Cardiorespiratory fitness was measured by a 2 km walk-test, strength by number of push-ups in 40 seconds, sit-ups in 40 seconds, handgrip strength left and right, and a jump-and-reach test. Best performance out of two trials was recorded. Coordination was measured by a test battery including standing on one leg with closed eyes, standing on one leg while moving the second leg in circles, and three test items with balls. For each test, a trained member of the staff judged the performance as well done, done, or failed. Flexibility was measured by a sit-and-reach test, trunk side bending, shoulder neck mobility, and hamstring and rectus femoris muscle extensibility. All test items were* Z*-transformed using the initial sample of 35-year-old men in 1992 as reference and their arithmetic mean built up a fitness index (*α* = .85). When more than 50% of the test items in coordination, flexibility, strength, or the 2 km walk-test were missing, no fitness index was calculated. This does not include logical zeroes as for example, during the sit-up test.

#### 2.2.3. Physical Health Status (HS)

Physical health status was assessed during a laborious health examination conducted by a practicing physician. After a detailed anamnesis the doctor made a diagnosis concerning orthopaedics, neurology, and cardiovascular system with the following results: 0 = “no limitations,” 1 = “minor limitations, not impacting daily life,” 2 = “limitations impacting daily life,” and 3 = “major limitations heavily impacting daily life.” A physical health status scale (0–9) was derived from the sum of the three limitation scales in orthopaedics, neurology, and the cardiovascular system.

#### 2.2.4. Socioeconomic Status (SES)

Based on methods for social structure analyses [27], the subjects were classified into four socioeconomic status categories using information about formal education and professional status. If participants were not working, the professional status of the life partner was used. Four categories were formed: low, mid/low, mid/high, and high SES.

### 2.3. Statistical Analysis

Statistical analysis was performed using SPSS Statistics 22.0. The function MIXED ML was used to conduct hierarchical linear models of PF and HS. All but the physical activity predictors and age were grand mean centered (GMC). Physical activity variables were untransformed with 0 meaning no physical activity and age was zeroed at its lowest value 28. This results in the constant term reflecting an average inactive person aged 28. Parameters in the models are age (zeroed at 28), age^2^ (zeroed at 28), sex (GMC), social status (GMC), athlete (no = 0; yes = 1), BMI (GMC), SA, HA, WRA, and every possible first-order interaction. A stepwise backwards technique was used including all parameters and interactions in an initial model. In each following step, the predictor or interaction term with the highest *p* value was eliminated followed by a rerun of the model. The final level of significance was set to *p* < .10 to compensate for the complexity of the models and because models with *p* < .05 showed a significant worse fit. The final model was reached when no parameter or interaction term showed a *p* value higher than .10.

## 3. Results

### 3.1. Descriptive Statistics

Descriptive statistics of SA, HA, WRA, PF, and HS data by sex and age group are shown in Table 2. *N* refers to the total number of observations during the four measurement points among the 723 participants.

SA shows a small increase from age group 28–40 to 41–50 and then slowly decreases over the observed course of lifespan. Contrary to SA, reported HA increases when the sample gets older and represents a large part of the physical activity in the elderly. The amount of WRA is relatively constant during the age of 28–60 and then decreases as people retire from work. Since most people at least spend 8 hours a day at work, the absolute numbers of spent MET-yours in WRA is larger than in SA or HA. Gender differences in physical activity favor men in all three types of PA.

PF shows expected gender differences favoring men and constantly declines with increasing age. However, as PF declines, the differences between men and women get smaller.

As PF decreases, the amount of detected health related limitations in the physical examination increases. Starting from only minor health related limitations in the age of 28–40, the health status of the sample declines over time up to a value of 3.25 standing for minor limitations in each, orthopaedics, neurology, and the cardiovascular system or major limitations in at least one of the considered areas.

### 3.2. Physical Fitness

The parameter estimation of the HLM modeling of PF is shown in Table 3. Numbers were rounded to two relevant ciphers.

An average inactive participant shows a fitness score of 93.74 (Table 3: “constant term,” for description, see statistics part). Sex is the strongest predictor of PF with men showing 7.00 *Z*-values higher PF than women. Squared age and age form the second important predictors of PF. Negative parameter estimates indicate an accelerating decline in PF with increasing age.

Irrespective of the amount of activity, participants who reported that they exercise show 1.50 *Z*-values higher PF than unsporting others (Table 3: “athlete”). In addition, PF increases about 0.052 *Z*-values per MET-h spent at SA. In comparison, PF increases about 0.013 *Z*-values per MET-h HA. WRA showed no significant influence on PF.

**Figure 2:**
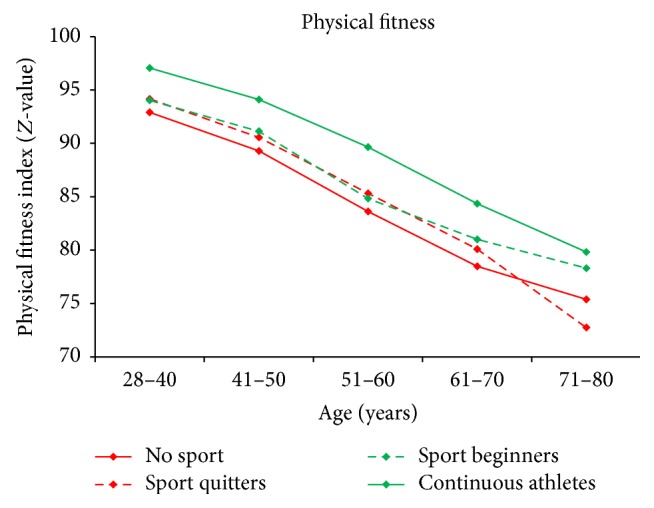
PF by age and sport activity. “No sport”: participant who continuously reported no SA; “sport quitters”: participants who reported SA at their first but not on their last examination; “sport beginners”: participants who reported no SA at their first but on their last examination; “continuous athletes”: participants who reported SA on each examination.

Figure 2 shows the development of PF over the course of the observed lifespan for four different exercise groups. Athletes show a higher PF than nonathletes in every age group. People who start exercising increase their PF whereas people who quit exercising lose PF. Interestingly, the initial value of PF for later quitters is lower than for continuous athletes.

Besides sex, age, and physical activity, SES and BMI are significant predictors of PF. Every increase in SES of one category shows an increase in PF by 0.91 *Z*-values. BMI is negatively associated with PF. A decrease of 0.18 *Z*-values in PF per BMI point was observed. Additionally, a positive estimate of the interaction parameter between age and BMI indicates an enhancing loss of PF per BMI with increasing age. However, a positive estimate of the interaction parameter between squared age and BMI shows that, in very high age groups, this relationship is reversed. However, with *p* = .08 and *p* = .09, respectively, those interaction terms are on the edge of the critical *p* value.

Finally, significant random effects of the constant term and BMI and age signalize significant amounts of intrapersonal variance in these parameters, respectively, the initial value of fitness performance.

### 3.3. Physical Health Status

The results of the HLM modeling of HS are shown in Table 4.

An average inactive participant aged 28 shows a HS score of 1.12 (constant term) indicating that the samples average participant in early adulthood shows rarely any lifestyle impacting health limitations. Age is the strongest predictor of HS with an increase of 0.053 in the limitation-score each year. Squared age was not a significant predictor indicating a linear age-related increase of the HS score. BMI is also a strong predictor of HS with an increase of 0.10 limitation-score-points per BMI point. Furthermore, SES is a significant predictor of HS with higher SES standing for a better HS.

**Figure 3:**
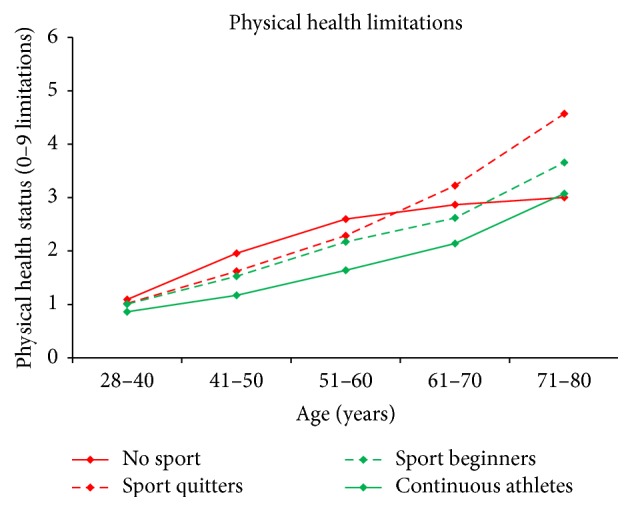
HS by age and sport activity. “No sport”: participant continuously reported no SA; “sport quitters”: participants who reported SA at their first but not on their last examination; “sport beginners”: participants who reported no SA at their first but on their last examination; “continuous athletes”: participants who reported SA on each examination.

Exercising in general shows significant positive effects on maintaining a good HS. The linear age-related loss in HS in early and midadulthood is nullified in athletes (age*∗*athlete: −0.068; age: +0.053). However, a significant, negative associated interaction term between squared age and athlete shows that athletes also lose HS and even faster at high ages.  Figure 3 shows the development of HS over the course of the observed lifespan for four different exercise groups.

The amount of SA shows no positive relationship with HS but is negatively associated when combined with high BMI values (SA*∗*BMI). HA showed a positive influence on HS, but only for males (sex*∗*HA). The relationship between WRA and HS is moderated by age. Starting from an increasing negative association between WRA and HS (age*∗*WRA), the relationship between WRA and HS reverses at higher ages and high amounts of WRA turn out to be a predictor for a good HS in older participants (age^2^*∗*WRA).

Besides moderating the effect of HA, the basic term of sex shows a slightly higher limitation-score for men. Contrary to PF, the constant term of the HS model has no significant random effect, indicating a more or less identical initial value between participants aged 28. However, a significant random effect of age shows that the slope of HS differs within participants.

## 4. Discussion

### 4.1. Major Findings

With increasing age, PF is decreasing and physical health limitations are increasing while SA is decreasing. These findings are consistent with numerous other studies [8, 28] and indicate that physical health parameters as well as SA decline with increasing age.

SA was positively associated with fitness and health with the exception of high amounts of SA at high BMI levels. Comparable amounts of habitual activity showed significantly smaller benefits and WRA showed no relationship to PF and only a low, inconsistent association with HS.

### 4.2. Influence of Different Types of PA on PF

Besides sex and age, SA turned out to be the most meaningful predictor for PF. Athletes possess a better PF than nonathletes in every age group and participants who started to exercise throughout the study gained, whereas participants who quitted exercise lost PF. This is in line with other studies about SA and PF [24, 29]. The amount of reported SA also showed a positive relationship to PF. The results confirm that, during every stage of life, SA is essential for keeping sufficient motor skills [30, 31].

The relationship between HA and PF differs from SA and PF. Even though the amounts of HA and SA were comparable in midadulthood and HA exceeded SA in the elderly, the relationship between HA and PF turned out to be considerable lower than between SA and PF. This may be due to the unsystematic character of HA and to its lower overall intensity. Few other studies did differentiate between HA and SA but those who did showed similar results. A recent study about aerobic fitness, exercise training, and HA showed that while exercise training enhances aerobic fitness, HA shows no meaningful relationship with fitness during youth [32].

The fact that WRA showed no positive effect on fitness has also been shown in previous studies. Recent results from a Canadian workplace management program with 4022 participants showed that the level of physical activity at work is not related to cardiorespiratory fitness or anthropometrics and cardiometabolic risk profile [33]. Other studies reported even negative effects of WRA on health parameters. Data from Gutiérrez-Fisac et al. [9] showed that high amounts of WRA are numerically associated with adiposity parameters. In this paper not presented analyses that differentiated fitness between motor performance abilities showed that WRA is negatively associated with flexibility, especially when people get older.

### 4.3. Influence of Different Types of PA on HS

Participants who reported to exercising showed a significant better HS than inactive. However, compared to the findings for PF, exercising showed less impact on HS. Even though many other studies do not differentiate between SA and HA, there is consensus about an overall positive relationship between leisure time PA and health parameters [34]. Interestingly our data showed that, starting from a higher level, the loss of HS in elderly athletes was higher than in nonathletes. This indicates that athletes cannot maintain their excellent HS for a lifetime and HS of athletes and nonathletes converges at higher ages. Further studies with high aged participants that investigate this finding are needed.

In addition to the positive relationship between exercising in general and HS, no positive relationship between the amount of SA and HS was observed. Contrariwise, high amounts of SA showed a negative relation with HS when combined with high values for BMI. This is in line with a study from Dorn et al. [35]. The authors report a positive relationship between PA and mortality risk but only for nonobese men and women. We conclude that high amounts and/or intensities of SA over a long period of time are not boundless healthy when talking about health limitations including orthopaedics and may even be noxious for people with high BMI scores when being not well executed. This thesis is supported by the data from Arem et al. [34] which shows an U-shaped relationship between PA and health with an increasing mortality risk at very high levels of PA. To date, most general statements from reviews about PA and HS suggest that PA is healthy at every BMI and in every stage of life [1]. This may be true for applied, supervised exercising but has to be reconsidered and further analyzed for daily life PA.

In our study, WRA was negatively associated with HS in early and midadulthood but a significant positive associated interaction between squared age and WRA indicates that, at older ages, people who report high amounts of WRA show a better HS. Whereas the negative association of WRA*∗*age is in line with other studies that find no [10, 33] or a negative association between WRA and HS [36, 37], the positive association between WRA*∗*age^2^ and HS could be due to the fact that, among older participants, only healthy ones are able to execute high amounts of WRA. A recent Scandinavian study showed that moderate and unfit people with high occupational physical activity are at higher risk for cardiovascular and all-cause mortality [38]. These findings about WRA are contrary to the early findings of Morris in his London Transport Workers Study [39]; however recent studies focus on a broader range of work related activities and also physical intense activities at work are included.

Many studies report that unsystematic PA like HA is not sufficient to achieve health outcomes [32]. In our study a significant interaction between HA and sex indicates that especially men benefit from HA. This could be due to higher intensities and higher amounts of HA among men which lead to successfully reaching the threshold for significant health effects in late adulthood.

### 4.4. Influence of Sociodemographic Variables and BMI

Men showed higher levels of PF than women, but a significant interaction term between sex and age showed that these differences decline with increasing age. Men showed a slightly worse HS compared to women. In addition, both SES and BMI showed a significant impact on PF and HS. SES and BMI turned out to be the most meaningful predictors of HS besides age. The influence of SES on HS is in line with other studies, showing a health benefit from higher SES [40, 41] but there are also studies who lack finding a consistent pattern of association between SES and health outcomes [42]. Lower values for PA and PF for residents with lower SES have been reported in numerous studies with adults [40] as well as adolescents [43, 44].

Interestingly, a pair of significant interactions between age and BMI and squared age and BMI concerning PF showed that the association between BMI and PF gets worse with increasing age but then turns in the elderly. A positive association between BMI*∗*age^2^ and PF indicates that, in late stage of life, a high BMI is a predictor for a better PF. The reason for this finding may lay in the phenomenon of sarcopenia, a decline in muscle mass in the elderly, which is indicated by a loss of BMI in late adulthood [45]. The fact that BMI does not differentiate between muscle and fat mass could be the reason for an observed, significant random effect of BMI on PF. Whereas in some individuals an increasing BMI due to an increase in muscle mass can go along with an increase in PF, in others, an increase in BMI due to body fat is negatively associated with PF.

### 4.5. Strength and Limitations of the Study

The main strengths of this study are the longitudinal data over a course of 18 years and the broadened view of PA, PF, and HS.

The average SA of about 10 MET-hours per week lies in the range of a representative German study that reports an average of 33.7% German residents with no SA, 40.9% with up to 2 h of SA, and 25.4% with more than 2 h of SA per week [22]. Though, the relatively high values for SA and HA among participants who aged 61–80 indicate a bias towards more active longitudinal participants. Nonresponder analyses showed that the difference between responders and nonresponders in HS, PF, and PA on their average last examination is under ten percent. We assume that the reason for a relatively low longitudinal bias is the distinct focus on health during the examination. We experienced that many unfit and relatively unhealthy participants remain in the sample because they use the opportunity of a detailed health check with an extensive talk to a practicing doctor.

In this study we draw conclusions about daily life PA and fitness and health from an observational longitudinal study because we believe that there is a lack of knowledge about effects of daily life PA on fitness and health. However, this design lacks a control group and a significant parameter estimate of PA in the HLM models does not stand for a causal effect from PA on HS or HF. From cross lagged panel designs we know that the relationship between PA and health is bidirectional [46] and in order to unravel clear dose-response principles we need random controlled studies [24]. However the aim of this study was to sensitize for the high impact of the context and content of PA and therefore our target was not to express causal effects in first line.

When methods of data collection are concerned the detailed assessment of PF and HS is a mentionable strength of this study. However, using a questionnaire to assess PA, variables tend to have low validity and reliability [47]. The used questionnaire showed a remarkable good reliability (test-retest after two weeks: *r* > .90 and Cronbach*ʼ*s alpha = .94) but little is known about criteria validity because there are no true objective criteria for assessing daily life PA in different settings. In order to obtain comparable data with accelerometers, participants would have to wear an accelerometer over the course of a broad time span (e.g., a year) and additionally keep a diary about the context of their activity. Defining time frames of different types of PA with the doubly labeled water method is even more striking and not feasible. Nevertheless, overestimating and response bias in PA could have influenced the reported levels of HA, SA, and WRA.

### 4.6. Conclusion

This study shows that different types of daily life physical activity differ in a meaningful way in their effects on fitness and health when a large lapse of time is observed. Whereas SA was positively associated with fitness and health with the exception of high amounts of SA at high BMI levels, comparable amounts of habitual activity showed only small benefits and WRA showed no or inconsistent effects. These findings show that the context and content, for example, adequate intensity, frequency, and execution, of PA are very important to utilize its benefits in daily life. The accelerated decline of HS in athletes as well as the high average of health limitations in sport quitters should be further examined.

## Figures and Tables

**Table 1:** Descriptive statistics of adult participants of the longitudinal study in Germany.

Variable	All participants	Females	Males
*N*	723	366	357
Number of observations	1681	828	853
Initial age (years)	44.73 ± 7.52	44.65 ± 7.51	44.81 ± 7.47
Average age (years)	48.67 ± 10.36	48.31 ± 10.28	49.02 ± 10.44
Average BMI (kg/m^2^)	26.11 ± 4.02	25.30 ± 4.28	26.91 ± 3.58
Athletes	62.3%	59.2%	65.4%
SES			
Low	6.9%	9.7%	4.0%
Low/mid	25.7%	24.4%	26.9%
Mid/high	37.1%	44.3%	30.0%
High	30.3%	21.5%	39.1%

**Table 2:** Mean (SD) values for physical activity, PF and HS of participants of the longitudinal study.

Age	sex	SA [MET-hours per week]	HA [MET-hours per week]	WRA [MET-hours per week]	PF [*Z*-score]	HS [scale units]
28–40 *N* = 460	m	11.69 ± 14.44	10.49 ± 14.19	47.21 ± 32.72	98.97 ± 3.95	1.02 ± 0.46
	f	8.66 ± 11.32	9.32 ± 19.96	30.59 ± 30.81	91.82 ± 3.60	0.84 ± 0.46
	∑	10.15 ± 13.03	9.89 ± 17.41	38.58 ± 32.78	95.40 ± 5.21	0.93 ± 0.47

41–50 *N* = 508	m	11.95 ± 13.72	12.26 ± 17.20	47.72 ± 32.11	95.15 ± 4.77	1.53 ± 0.63
	f	10.94 ± 12.99	11.11 ± 17.60	38.75 ± 32.24	89.30 ± 4.10	1.37 ± 0.59
	∑	11.47 ± 13.37	11.70 ± 17.39	43.41 ± 32.45	92.34 ± 5.29	1.45 ± 0.62

51–60 *N* = 468	m	9.18 ± 14.01	18.28 ± 24.25	43.62 ± 37.88	88.85 ± 5.59	2.15 ± 0.90
	f	8.12 ± 11.60	15.10 ± 21.59	33.16 ± 33.47	84.05 ± 4.63	2.08 ± 0.85
	∑	8.65 ± 12.87	16.69 ± 22.99	38.43 ± 36.10	86.49 ± 5.67	2.12 ± 0.87

61–70 *N* = 172	m	8.90 ± 11.60	31.39 ± 38.30	12.78 ± 26.29	83.21 ± 5.53	2.69 ± 1.18
	f	8.45 ± 11.37	20.88 ± 24.95	19.96 ± 35.83	79.08 ± 5.06	2.60 ± 1.02
	∑	8.69 ± 11.46	26.25 ± 32.81	16.22 ± 31.35	81.41 ± 5.69	2.65 ± 1.11

71–80 *N* = 48	m	10.48 ± 11.95	28.65 ± 25.92	4.21 ± 16.06	77.11 ± 5.58	3.41 ± 1.16
	f	5.51 ± 8.79	29.64 ± 23.24	9.67 ± 24.28	76.07 ± 3.89	2.96 ± 1.18
	∑	8.57 ± 11.01	29.02 ± 24.70	6.32 ± 19.56	76.74 ± 5.01	3.25 ± 1.17

**Table 3:** THLM model for physical fitness of 723 participants in the Bad Schönborn study.

Fixed effects (in order of influence according to *F*-value)					
Parameter	Estimate	[95% CI]	SE	*F*	*p*

Constant term	93.74	[92.96–94.52]	0.40	55611.18	<.01
Sex (if male)	7.00	[5.86–8.14]	0.58	145.04	<.01
Age^2^ (per year^2^)	−0.0072	[−0.0095–−0.0049]	0.0012	37.75	<.01
Age (per year)	−0.20	[−0.28–−0.12]	0.041	23.09	<.01
Athlete (if yes)	1.50	[0.83 to 2.17]	0.34	19.31	<.01
SA (per MET-h)	0.052	[0.029–0.076]	0.052	19.12	<.01
SES (per enhancing social stratum)	0.91	[0.47–1.35]	0.22	16.54	<.01
Age*∗*sex	−0.083	[−0.145–−0.020]	0.032	6.66	.01
BMI (per BMI point)	−0.18	[−0.36–−0.01]	0.094	3.51	.06
HA (per MET-h)	0.013	[−0.001–0.026]	0.067	3.47	.06
Age*∗*BMI	−0.018	[−0.362–0.009]	0.010	3.11	.08
Age^2^*∗*BMI	0.00048	[−0.00008–0.00100]	0.00029	2.81	.09

Random effects					

Parameter	Estimate	[95% CI]	SE	Wald *Z*	*p*

Constant term	14.23	[11.23–18.03]	1.72	8.27	<.01
BMI	0.12	[0.04–0.35]	0.06	1.81	.07
Age	0.0070	[0.0021–0.0223]	0.0041	1.69	.09

Model fit					

Correlation between predicted and measured values: *r* = .94 −2 Log-Likelihood: 8207.19					

**Table 4:** HLM Model for the health status of 723 participants in the Bad Schönborn study.

Fixed effects					
Parameter	Estimate	[95% CI]	SE	*F*	*p*

Constant term	1.12	0.87–1.51	0.11	69.69	<.01
Age (per year)	0.053	0.039–0.067	0.007	53.26	<.01
BMI (per BMI point)	0.10	0.08–0.13	0.014	53.17	<.01
SES (per enhancing social stratum)	−0.20	−0.29–−0.11	0.05	17.60	<.01
SA*∗*BMI	0.0040	0.0019–0.0061	0.0011	14.05	<.01
Athlete*∗*BMI	−0.084	−0.131–−0.037	0.024	12.31	<.01
Age*∗*athlete	−0.068	−0.104–−0.033	0.018	14.28	<.01
Age^2^*∗*athlete	0.0016	0.00060–0.00260	0.0005	9.86	<.01
Age*∗*WRA	0.00077	0.00017–0.00260	0.00031	6.33	.01
Age^2^*∗*WRA	−0.000025	−0.000044–−0.000006	0.000010	6.49	.01
Sex*∗*HA	−0.0077	−0.015–−0.006	0.0077	4.57	.03
Sex (if male)	0.18	−0.01–0.37	0.096	3.66	.06
WRA	−0.0036	−0.0082–0.0011	0.0024	2.21	.14^*∗*^
SA	0.0034	−0.0033–0.0102	0.0035	0.99	.32^*∗*^
HA	0.0012	−0.0024–0.0048	.0018	0.45	.50^*∗*^
Athlete	−0.033	−0.35–0.29	0.16	0.04	.84^*∗*^

Random effects					

Parameter	Estimate	[95% CI]	SE	Wald *Z*	*p*

Age	0.0011	[0.0021–0.0223]	0.0002	5.51	<.01

Model fit					

Correlation between predicted and measured values: *r* = .71 −2 Log-Likelihood: 4736.91					

^*∗*^Basic terms of parameters have to be included when interactions with them are significant.

## Abbreviations

PA: Physical activity
PF: Physical fitness
HS: Physical health status
HLM: Hierarchical linear modeling
SA: Sports activity
HA: Habitual activity
WRA: Work related activity
SES: Socioeconomic status
BMI: Body mass index.
